# Gene family expansions in Antarctic winged midge as a strategy for adaptation to cold environments

**DOI:** 10.1038/s41598-022-23268-9

**Published:** 2022-10-29

**Authors:** Heesoo Kim, Han-Woo Kim, Jun Hyuck Lee, Joonho Park, Hyoungseok Lee, Sanghee Kim, Seung Chul Shin

**Affiliations:** 1 Division of Life Sciences, Korea Polar Research Institute (KOPRI), Incheon, 21990 Republic of Korea; 2 Animal and Plant Research Department, Nakdonggang National Institute of Biological Resources (NNIBR), Sangju-si, Republic of Korea; 3 Research Unit of Cryogenic Novel Material, Korea Polar Research Institute, Incheon, 21990 Republic of Korea; 4 Department of Polar Sciences, University of Science and Technology, Incheon, 21990 Republic of Korea; 5 Department of Fine Chemistry, Seoul National University of Science and Technology, Seoul, Republic of Korea

**Keywords:** Evolutionary biology, Genome evolution

## Abstract

*Parochlus steinenii* is the only flying insect native to Antarctica. To elucidate the molecular mechanisms underlying its adaptation to cold environments, we conducted comparative genomic analyses of *P. steinenii* and closely related lineages. In an analysis of gene family evolution, 68 rapidly evolving gene families, involved in the innate immune system, unfolded protein response, DNA packaging, protein folding, and unsaturated fatty acid biosynthesis were detected. Some gene families were *P. steinenii-*specific and showed phylogenetic instability. Acyl-CoA delta desaturase and heat shock cognate protein 70 (Hsc70) were representative gene families, showing signatures of positive selection with multiple gene duplication events. Acyl-CoA delta desaturases may play pivotal roles in membrane fluidity, and expanded Hsc70 genes may function as chaperones or thermal sensors in cold environments. These findings suggest that multiple gene family expansions contributed to the adaptation of *P. steinenii* to cold environments.

## Introduction

Antarctica is one of the most extreme environments for life. The continent is isolated from the Southern Hemisphere by the Southern Ocean and atmospheric circulation, and most of the area, over 99.6%, is covered by snow or ice. As a result, Antarctic terrestrial biodiversity is low and ecosystems restricted to low-altitude coastal regions of the Antarctic Peninsula, where seasonal snowmelt occurs^1,2^. Among the estimated 5.5 million species of insects worldwide^3^, only three species, *Belgica antarctica*, *Eretmoptera murphyi*, and *Parochlus steinenii,* have been recorded in the maritime Antarctic region^4^. These species belong to the family Chironomidae and overwinter in their larval stage. *B. antarctica*, which is native to Antarctica, and *E. murphyi*, a well-established invasive species, are freeze-tolerant wingless midges. *P. steinenii* has wings and is not freeze-tolerant but shows limited cold tolerance with a lower lethal temperature of around − 3 °C^4–6^; accordingly, is expected to have different strategies for cold adaptation compared to the other two species.

The genome of *B. antarctica* has been sequenced and transcriptome analyses of larvae have provided insight into its adaptation to the extreme environment of Antarctica^4,7,8^. However, genetic studies of *P. steinenii* are lacking. We previously reported the draft genome of *P. steinenii*^9^ as well as an improved genome assembly and annotation method using nanopore sequencing and genome polishing tools^10^*.* The genome of *P. steinenii* is about 145.37 megabases (Mb), which is about 46 Mb larger than the compact genome of *B. antarctica* (99 Mb)^7^. In our previous study, focusing on the improvement of assembly using nanopore reads, we did not investigate the mechanisms underlying *P. steinenii* cold-adaptation. In the present study, we aimed to use high-quality assembly and annotation for comprehensive genomic analyses using recent tools, and perform comparative genomic analyses of *P. steinenii* and closely related lineages to elucidate these mechanisms.

## Results

### Genome assembly and annotation

In this study, a genome assembly of *P. steinenii* with a superior quality to that of the previous reported genome was obtained (Table 1). The final assembled genome of *P. steinenii* consisted of 56 scaffolds with a total size of 144.22 Mb, an N50 value of 7 Mb, and a maximum scaffold length of 17.38 Mb. The genome was similar in size to the previously reported genome (145.37 Mb)^10^ but had fewer scaffolds, higher N50 values, and a longer maximum scaffold length.

**Table 1:** Comparison of assembly and annotation statistics for the new and previously reported *P. steinenii* genomes.

	Initial assembled genome^10^	Final assembled genome (this study)
**Gene assembly**		
Assembler	SMARTdenovo^11^	NextDenovo (v.2.4.0)
Polishing	Nanopolish (v.0.10.1) + Pilon (v.1.22)	NextPolish (v.1.3.1)
Assembly size (Mb)	145.37	144.22
Scaffolds (#)	162	56
Scaffold N50 (Mb)	1.989	6.945
Maximum scaffold length (Mb)	9.64	17.38
BUSCO completeness (%) of genome (Eukaryote *odb9*/ Insecta *odb9*)	98.7/98.4	98.9/98.6
**Gene annotation**		
Genes (#)	11,938	12,461
Gene density (% of genome covered by genes)	41.5	41.2
Average intron length (bp)	226	364
Average exon length (bp)	392	454
Repeat content (%)	11.47	12.56
BUSCO completeness (%) of gene set (Eukaryote *odb9*/ Insecta *odb9*)	88.8/90	95.7/95.8

**Figure 1:**
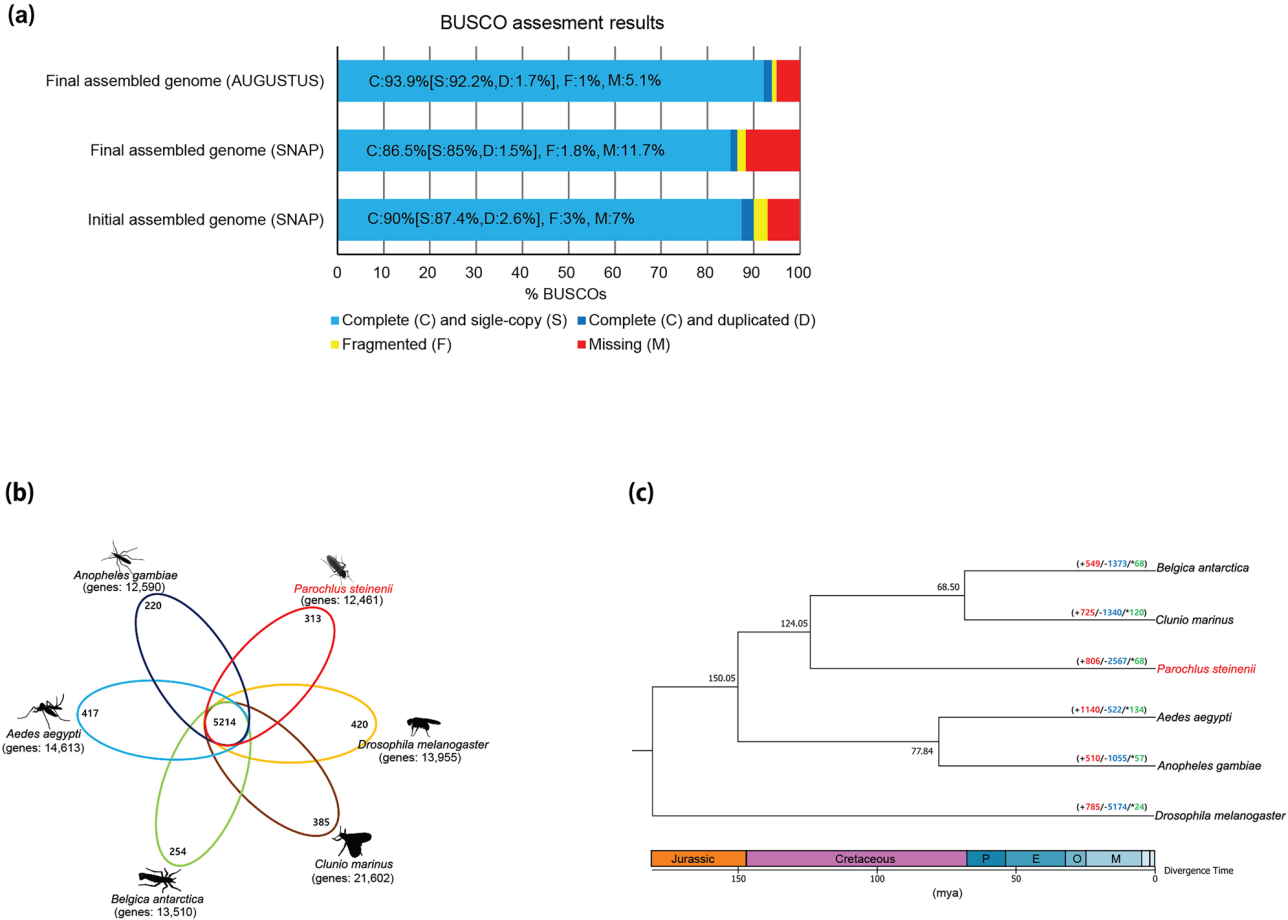
BUSCO assessment of gene set and Comparison of *P. steinenii* protein-coding genes. (**a**) Completeness assessment of gene sets using BUSCO. The BUSCO dataset of the Insecta *odb9* were used to assess the gene sets predicted from the initial assembled genome using SNAP and the gene sets from the final assembled genome using SNAP or AUGUSTUS. (**b**) Venn diagram of orthologous gene families among six dipteran genomes. (**c**) Lineage-specific gene gains and losses in six dipteran genomes. The number of gene gains, losses, and the rapidly evolving gene families were marked in red, blue, and green, respectively. E, M, O, and P denote Eocene, Miocene, Oligocene, and Paleocene, respectively. The aligned sequences of orthologous gene families were used to construct a phylogenetic tree using FastTree (v.2.1.10) and divergence time between species was inferred using TimeTree.

The assembled genome of *P. steinenii* was annotated using the MAKER annotation pipeline based on protein homology and EST data. A total of 12,461 protein-coding genes were predicted in the assembled genome, and 10,985 protein-coding genes were assigned functions by searches against EggNOG (Supplementary Data 1). Repeat prediction using a de novo RepeatModeler repeat library confirmed that 12.56% of the assembled genome was repeat sequences. Additionally, 492 tRNAs were predicted (Supplementary Table 1). The number of genes, and average intron and exon length were slightly increased in the new assembly compared to the previously reported genome (Table 1). In particular, BUSCO analyses with two datasets (Eukaryote *odb9* and Insecta *odb9*) indicated that the gene set completeness using AUGUSTUS was markedly better than that of the previously reported genome (Table 1). These results indicate that the quality and completeness of the assembled genome were sufficiently high to ensure reliable subsequent genomic analyses (Fig. 1a).

### Evolution of gene families in *P. steinenii*

Protein sequences of *Aedes aegypti* (GCF_002204515, NCBI), *Anopheles gambiae* (GCF_000005575, NCBI), *B. antarctica* (ASM77530v1, Ensemble), *Clunio marinus* (GCA_90005825, NCBI), and *Drosophila melanogaster* (GCF_000001215, NCBI) were used for gene family analysis, and isoforms were discarded prior to analysis. The total proteins of the six dipteran species were categorized into 13,482 gene families using OrthoVenn2 (v.2019-05-26)^12^. A total of 5,214 gene families were shared by the six dipteran species, whereas 313 gene families were specific to *P. steinenii* (Fig. 1b and Supplementary Data 2). In a phylogenetic analysis based on shared orthologous gene families from the six dipteran species, the estimated divergence time between *P. steinenii* and other Chironomidae species (*B. antarctica* and *C. marinus*) was about 124.05 million years ago (Fig. 1c). In the *P. steinenii* lineage, 806 significantly expanded gene families, 2,567 significantly contracted gene families, and 68 significantly rapidly evolving gene families were identified (Supplementary Table 2).

**Figure 2:**
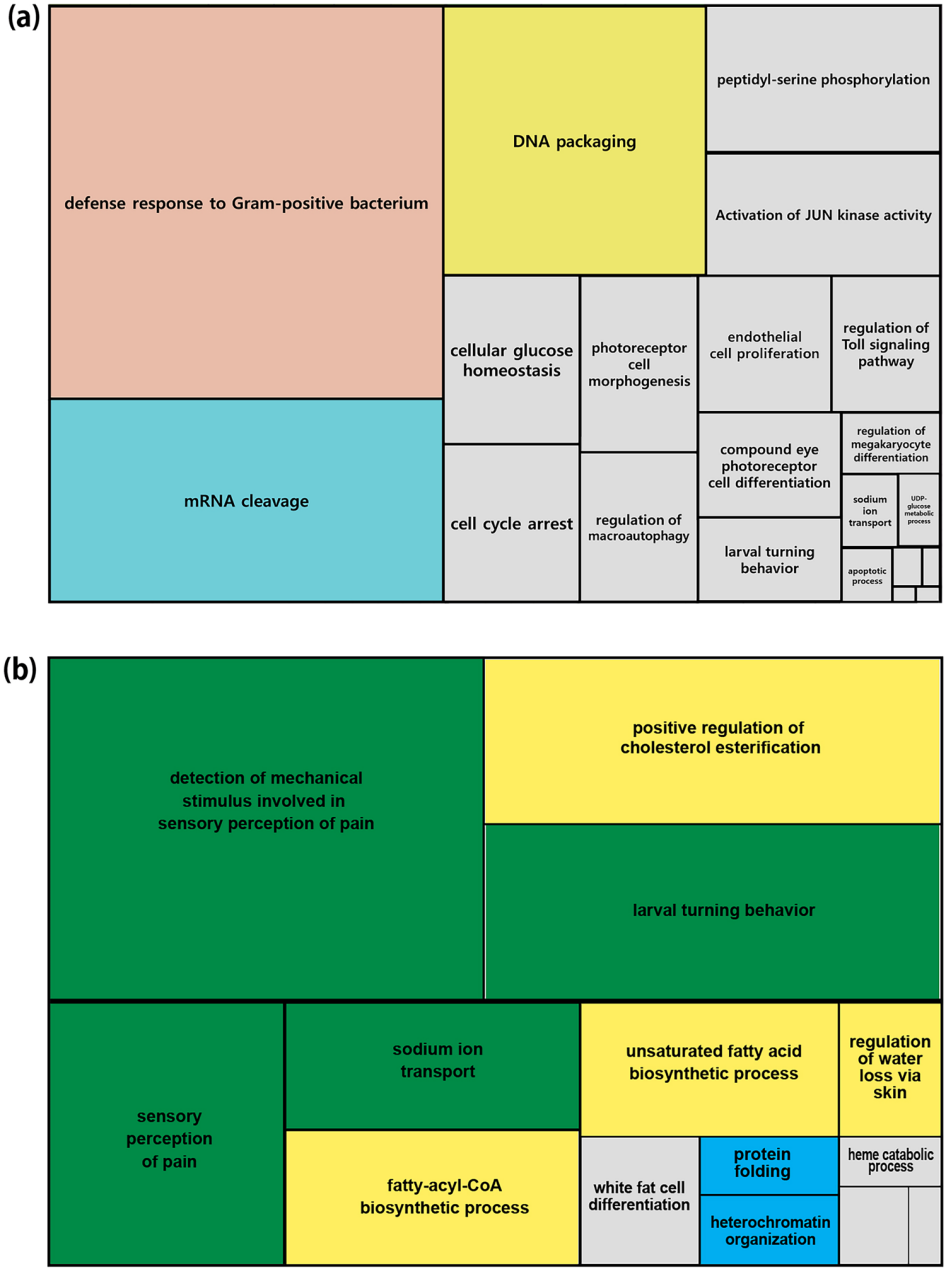
Enriched GO term of rapidly evolving gene families in *P. steinenii*. (**a**) TreeMap summarized from the redundant GO terms using REVIGO for all GO terms linked to rapidly evolving gene families. (**b**) GO terms linked to 25 *P. steinenii*-specific gene families. Enriched biological process terms were visualized using REVIGO. The terms “detection of mechanical stimulus involved in sensory perception of pain,” “larval turning behavior,” “sensory perception of pain,” and “sodium ion transport” were related to expansions of pickpocket protein 28 gene family (group51) and group6074. “Positive regulation of cholesterol esterification,” “fatty-acyl-CoA biosynthetic process”, and “unsaturated fatty acid biosynthetic process” were related to the expanded acyl-CoA delta desaturase gene family (group1599), and “protein folding” was related to the expanded Hsc70 gene family (group513).

To infer functions of rapidly evolving gene families, we performed GO enrichment analyses. The rapidly evolving gene families in *P. steinenii* were significantly enriched in GO categories related to the defense response to Gram-positive bacterium, mRNA cleavage, and DNA packaging (Fig. 2a).

### *P. steinenii*-specific gene family expansion

We identified 25 rapidly evolving gene families that were found only in *P. steinenii*. The function and amino acid sequence of these gene families may be similar to other orthologous gene families of the six dipteran species but these 25 rapidly evolving gene families were divided into other gene families (Supplementary Table 2). In an analysis of over-represented GO terms for *P. steinenii*-specific gene families, we found enrichment for various biological processes, such as sensory perception of pain, unsaturated fatty acid biosynthetic process, and protein folding (Fig. 2b). These enriched GO terms were derived from the expanded acyl-CoA delta desaturase, heat shock cognate protein 70 (Hsc70), and pickpocket protein 28 gene families, respectively. Among them, the acyl-CoA delta desaturase gene family (group1599) and Hsc70 (group513) families may facilitate *P. steinenii* adaptation to the cold environments.

**Figure 3:**
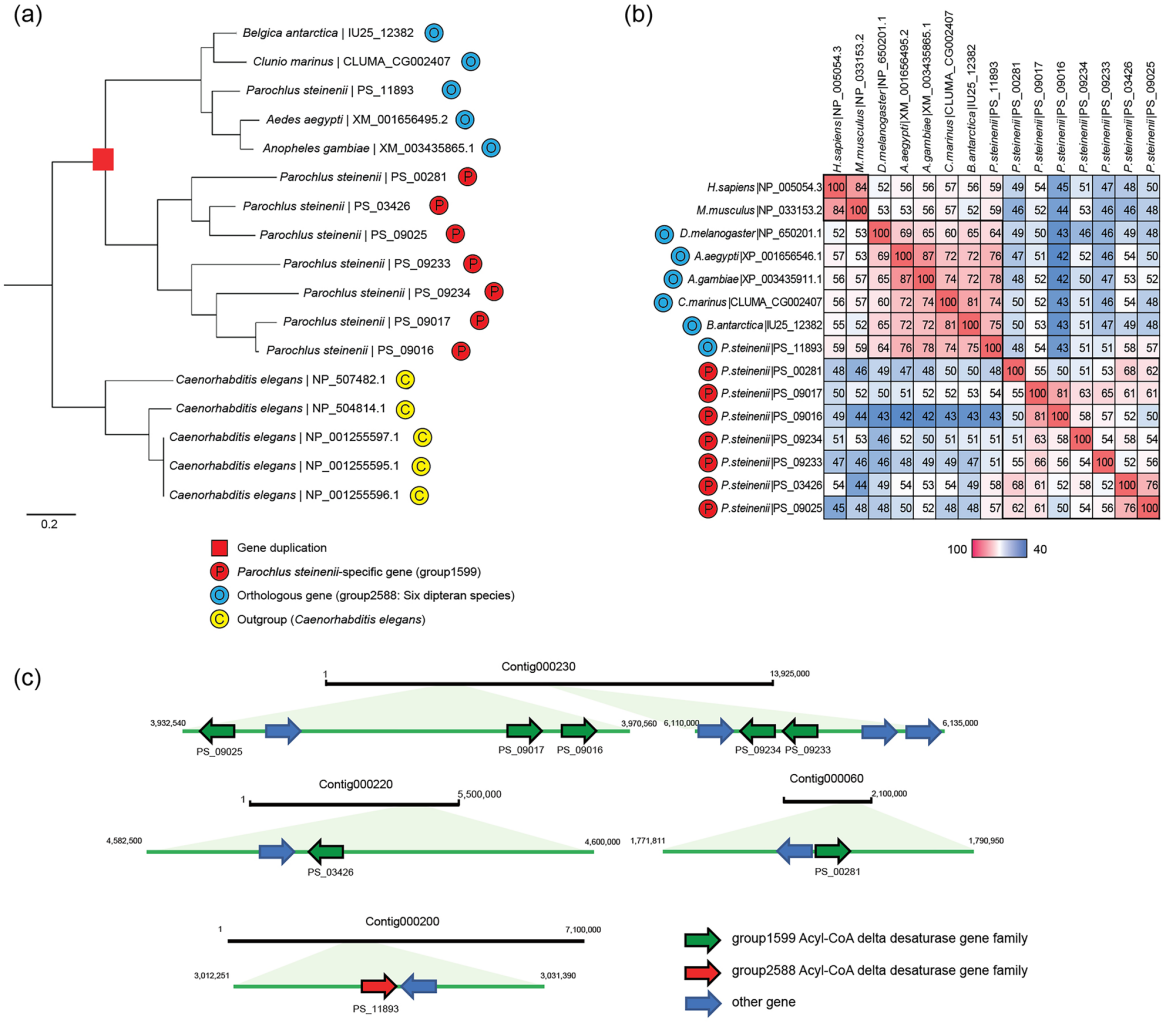
The expanded acyl-CoA delta desaturase gene family of *P. steinenii.* (**a**) Phylogenetic analysis of acyl-CoA desaturase genes in the *P. steinenii*-specific gene family (group1599) and orthologous gene family (group2588). Orthologous gene families in *C. elegans* were designated as outgroups. Numbers at nodes are bootstrap values from 1000 replicates and GenBank accession numbers are indicated at the ends of branches. (**b**) Amino acid sequence similarity matrix of acyl-CoA delta desaturase genes. *P. steinenii*-specific acyl-CoA delta desaturase (group1599), orthologous groups of acyl-CoA delta desaturase among in six insect species (group2588), stearoyl-CoA desaturase NP_005054.3 (*Homo sapiens*), and NP_033153.2 (*Mus musculus*) were used for the similarity matrix. Percent identities were calculated using the result of BlastP. (**c**) Expanded acyl-CoA delta desaturase gene families identified in the *P. steinenii* genome. *P. steinenii*-specific gene families are indicated by green arrows and orthologous genes are indicated by red arrows. Other genes are indicated in blue arrows. Each arrow indicates a gene orientation (5′ → 3′).

**Figure 4:**
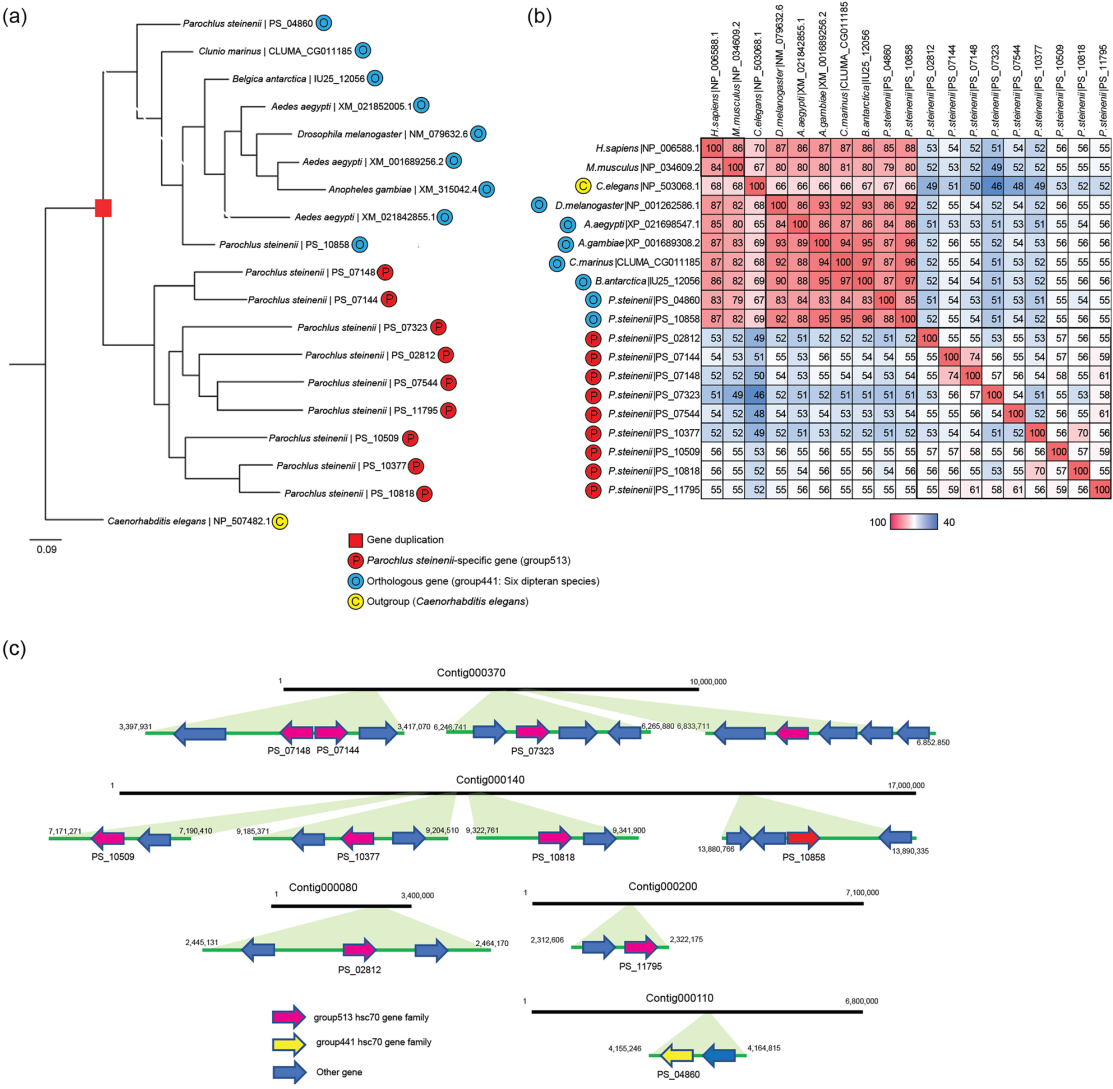
The expanded Hsc70 gene family of *P. steinenii.* (**a**) Phylogenetic analysis of Hsc70 genes in the *P. steinenii*-specific gene family (group513) and orthologous gene family (group441). Orthologous gene families in *C. elegans* were designated as outgroups. Numbers at nodes are bootstrap values from 1000 replicates and GenBank Accession numbers are indicated at the ends of branches. (**b**) Amino acid sequence similarity matrix of Hsc70 genes. *P. steinenii*-specific Hsc70 (group513), orthologous groups of Hsc70 genes among in six insect species (group441), NP_006588.1 (*H. sapiens*), NP_034609.2 (*M. musculus*), and NP_503068.1 (*C. elegans*) were used for the similarity matrix. Percent identities were calculated using the result of BlastP. (**c**) Expanded Hsc70 gene families identified in the *P. steinenii* genome. *P. steinenii*-specific gene families are indicated by pink arrows and orthologous genes are indicated by yellow arrows. Other genes are indicated in blue arrows. Each arrow indicates a gene orientation (5′ → 3′).

A maximum likelihood phylogenetic analysis of these two gene families along with orthologous gene families of the six dipteran species and *Caenorhabditis elegans* was conducted. *P. steinenii*-specific gene families formed an independent clade with low similarity to orthologous gene families in the six dipteran species (Figs. 3a, 4a). We speculated that the sequence divergence resulted from positive selection by gene duplication. To confirm this, we calculated the *d*_N_/*d*_S_ ratios (i.e., *ω*) for these gene families (Table 2). Since the *p* value for H_1_ (i.e., divergence between *P. steinenii-*specific and orthologous gene families) over H_0_ was less than 0.001, it can be inferred that the *ω* value of the *P. steinenii*-specific gene family was different from that of the orthologous gene family. To find potential coding sites under positive selection for acyl-CoA delta desaturase and Hsc70, we used Bayes empirical Bayes (BEB) (Table 3). Two and thirteen amino acid sites were under positive selection in acyl-CoA delta desaturase and Hsc70, respectively, with strong support for the BEB posterior probability (> 99%).

**Table 2:** Selective pressures in acyl-CoA delta desaturase and Hsc70.

	ω_A0_	ω_A1_	ω_P0_	ω_P1_	lnL	LRT	*p *value
**Acyl-CoA delta desaturase**							
H_0_ (ω_A0_ = ω_A1_ = ω_P1_ = ω_P0_)	0.0827	0.0827	0.0827	0.0827	− 6020.116	–	–
H_1_ (ω_A0_ = ω_A1_ ≠ ω_P1_ = ω_P0_)	0.0595	0.0595	0.1168	0.1168	− 6012.082	16.068	< 0.001
**Hsc70**							
H_0_ (ω_A0_ = ω_A1_ = ω_P1_ = ω_P0_)	0.05	0.05	0.05	0.05	− 19,093.435	**–**	**–**
H_1_ (ω_A0_ = ω_A1_ ≠ ω_P1_ = ω_P0_)	0.0316	0.0316	0.1159	0.1159	− 19,016.043	77.393	< 0.001

The null hypothesis (H0) and the alternative hypothesis (H1) were defined as the case where *d*_N_/*d*_S_ (ω) values are identical in *P. steinenii*-specific and orthologous gene families and when ω differs between *P. steinenii*-specific and orthologous gene families, respectively. The ω (ω_A0_, ω_A1_, ω_P1_, and ω_P0_) indicates the *d*_N_/*d*_S_ ratio for the corresponding branch (Supplementary Fig. 3). All *p *values were calculated using a log-likelihood (lnL) values and likelihood ratio test (LRT) and adjusted false discovery rate (FDR).

**Table 3:** Branch-site test of positive selection on *P. steinenii*-specific acyl-CoA delta desaturase and Hsc70.

		Site class^a^					lnL	LRT	*p *value
		0	1		2a	2b			
**Acyl-CoA delta desaturase**									
Null model A^b^									
Proportion^c^		0.821	0.044		0.129	0.007	− 6002.098	–	–
Background ω^d^		0.084	1.000		0.084	1.000			
Foreground ω^d^		0.084	1.000		1.000	1.000			
Model A^b^									
Proportion		0.831	0.044		0.119	0.006	− 5997.198	9.8	0.002
Background ω		0.085	1.000		0.085	1.000			
Foreground ω		0.085	1.000		135.506	135.506			
Positively selected sites: 8 Q**, 10 S*, 11 G*, 17 F*, 54 N**, 77 V*, 118 A*, 123 F*, 137 G*									
**Hsc70**									
Null model A									
Proportion	0.775		0.053		0.160	0.011	− 19,037.180	–	–
Background ω	0.0574		1.000		0.0574	1.000			
Foreground ω	0.0574		1.000		1.000	1.000			
Model A									
Proportion		0.795		0.056	0.139	0.010	− 19,026.171	11.008	< 0.001
Background ω		0.059		1.000	0.059	1.000			
Foreground ω		0.059		1.000	7.343	7.343			
Positively selected sites: 18 F*, 28 F**, 36 I**, 40 D*, 52 Q*, 54 Q*, 55 N**, 203 K*, 208 L**, 223H*, 242 S**, 250 K**, 265 Q**, 307 A*, 358 C**, 373 I*, 375 T*, 421 S**, 423 K*, 446 E**, 463 L**, 470 M* 476 T**, 479 N**									

All *p *values were calculated using InL and LRT values and adjusted using the FDR (**p-value < 0.01 and *p value < 0.05).

^a^Branch-site model for both gene families is run under the assumption of four site classes: class 0 = sites under negative selection in all gene family branches; class 1 = sites evolving neutrally in all gene family branches; class 2a = sites positively selected at *P. steinenii*-specific gene family branches, but negatively selected at other orthologous gene family branches; class 2b = sites positively selected on *P. steinenii*-specific gene family branches, but neutrally evolving on other orthologous gene family branches.

^b^The branch-site model are defined two types of model: Model A allowing positive selection along *P. steinenii*-specific gene family branches; Null Model A allowing neutral evolution and negative selection.

^c^The proportion indicates the fraction of sites within the gene family that belongs to each of the four site classes.

^d^Foreground and background ω are the dN/dS ratio for the *P. steinenii*-specific gene family branches and all other orthologous gene family branches, respectively.

When comparing the predicted gene structures of the expanded acyl-CoA delta desaturase genes and orthologues against that of rat *SCD1*, the two acyl-CoA delta desaturase gene families in orthologues completely shared eight histidine site for the primary coordination sphere of the dimetal unit essential to the function of rat SCD1 on gene structure, and did not appear to be inferior in function compared to SCD1 (Supplementary Fig. 1). However, when comparing the amino acid sequences of the expanded acyl-CoA delta desaturase orthologous group, similarities ranged from 43 to 58% (Fig. 3b), and half of CoA binding sites were different (Supplementary Fig. 1). When compared to rat SCD1, the expanded acyl-CoA delta desaturase gene family had amino acid differences (Tyr104—Glycine, Alanine, Isoleucine, and Methionine; Ala108—Isoleucine and Valine) at the Tyr104 and Ala108 positions^13^, which affect the length of the acyl chain in the substrate. In the case of the expanded Hsc70 gene family, when comparing *P. steinenii* Hsc70 and orthologous sequences, amino acid similarities ranged from 51 to 55% (Fig. 4b). The nucleotide-binding domain (NBD) in the substrate-binding domain was highly conserved; however, the substrate-binding domain (SBD) of *P. steinenii*-specific Hsc70 genes had a relatively lower similarity than that of the NBD and lacked the EEVD motif and lid comprising the G/P-rich C-terminal region^14^ (Supplementary Fig. 2).

### Patterns of gene duplication in the *P. steinenii* genome

As shown in Table 4, dispersed duplication events in the *P. steinenii* genome appeared to play an important role in gene family evolution, accounting for 47.60% of duplication events, similar to the frequency in the *B. antarctica* genome (Supplementary Table 3). In the rapidly evolving gene families, tandem duplications were most frequent in the *P. steinenii* genome. The expanded acyl-CoA delta desaturase and Hsc70 gene families mainly exhibited tandem and dispersed duplications, respectively (Figs. 3c, 4c).

**Table 4:** Gene duplication in the *P. steinenii* genome.

	Singleton	Dispersed duplication	Proximal duplication	Tandem duplication	WGD/segmental duplication
Total gene families	3,136 (31.76%)	**4700** **(47.60%)**	567 (5.74%)	1,403 (14.21%)	67 (0.68%)
Rapidly evolving gene families	0 0.00%	337 (37.78%)	141 (15.81%)	**388** **(43.50%)**	26 (2.91%)
Acyl-CoA delta desaturase	0 (0.00%)	1 (14.28%)	1 (14.28%)	**5** **(71.44%)**	0 0.00%
Hsc70	0 (0.00%)	**7** **(55.56%)**	1 (22.22%)	1 (22.22%)	0 (0.00%)

Proportions according to origins of the duplicate gene categories are shown in parentheses and the highest result in each row is indicated in bold.

## Discussion

Rapidly evolving gene families with high divergence among closely related species tend to be more closely related to adaptation than other genes^15^. Based on this, we speculated that the rapidly evolving gene families in the *P. steinenii* genome contributed to survival and adaptation to the harsh Antarctic environment. Unlike *B. antarctica*, which has a compact genome size with few repetitive elements and reduced intron length due to an extreme environment^7^, *P. steinenii* has an estimated genome size (144.2 Mb) similar to that of *Drosophila melanogaster* (139.5 Mb), suggesting that it used different strategies from those of *B. antarctica*.

Prior to gene family analysis, we attempted to improve the completeness of genome assembly and gene prediction using the latest tools. We changed the assembler from SMARTdenovo to NextDenovo (v.2.4.0). For polishing, NextPolish was used instead of Nanopolish (v.0.10.1) and Pilon (v.1.22). The assembly statistics were slightly improved by the change of assembler. However, as shown in Fig. 1a, the effects of NextPolish on BUSCO assessment of the gene set were unclear although it was reported to correct insertions and deletions better than Pilon^16^. The gene set completeness of the final assembled genome using SNAP (v.2006-07-28) for ab initio gene prediction in the MAKER annotation pipeline was lower than that of the initial assembled genome. The improvement of the gene set completeness of the final assembled genome appears to be due to AUGUSTUS (v.3.2.3).

The major biological processes identified in a GO enrichment analysis of the rapidly evolving gene families in *P. steinenii* (i.e., defense response to Gram-positive bacterium, mRNA cleavage, and DNA packaging; Fig. 2a) provide insight into the mechanisms underlying survival in cold environments. Among rapidly evolving gene families in the *P. steinenii* genome, several serine protease-related gene families (e.g., serine protease, serine protease Persephone, serine protease trypsin, and venom serine protease) were involved in the defense response to Gram-positive bacterium. Innate immune systems act as the first defense barrier against a variety of infections by rapidly recognizing foreign threats^17^. The Toll pathway is a major signaling pathway for the robust innate immune response of insects and is mainly responsible for the recognition of Gram-positive bacteria, fungi, and virulence factors and the production of certain antimicrobial peptides secreted into the insect hemolymph^18,19^. When infected with gram-positive bacteria, Toll receptor is activated by a serine protease cascade, leading to the cleavage of Spaetzle. Cleaved Spaetzle then binds to the Toll receptor^20^, generating the Toll-induced signaling complex, which is composed of MyD88, Tube, and Pelle. Signaling from the Toll-induced signaling complex is transmitted to Cactus, ultimately leading to the nuclear translocation of Dorsal-related immunity factor (DIF) and activation of genes encoding antimicrobial peptides^21,22^. Immune system function is critical for surviving bacterial infection and regulate gut bacteria, and it has been reported to be related to cold stress^23^. In *Drosophila*, immune activation by cold was suggested as a mechanism to compensate for the reduced immune function due to cold^24^, and it is also reported that environmental temperature significantly effect on the immune response and on the energetic costs of immunity in *Tenebrio molitor* larvae^25^.

In *P. steinenii*, rapidly evolved gene families related to the immune response, including modular serine protease, Persephone like protein, and phenoloxidase-activating factor, might be required in the harsh environment to maintain stable immune function. In addition, inositol-requiring enzyme (IRE1) and serine/threonine-protein kinase/endoribonuclease IRE1α families were associated with mRNA cleavage and the unfolded protein response (UPR), a critical adaptive function in cold environments identified in a previous study of the *P. steinenii* genome^9^. Excessive cellular disturbances (e.g., malnutrition and hypoxia) disrupt protein folding in the endoplasmic reticulum (ER), leading to the accumulation of misfolded proteins (known as ER stress). When these misfolded proteins accumulate and exceed a certain threshold, the UPR, a signal transduction pathway, is activated to restore homeostasis^26^. IRE-1 and serine/threonine-protein kinase/endoribonuclease IRE1α activate the UPR and maintain ER homeostasis^27,28^. The cold environment of Antarctica can also cause cellular disturbances, and the IRE-1 and serine/threonine-protein kinase/endoribonuclease IRE1α families may have rapidly adapted for increased UPR activateion to prevent misfolded proteins and restore homeostasis. The rapid evolution of histone-related gene families (e.g., histone H3 and histone H4) involved in DNA packaging was also detected in the *P. steinenii* genome, and these genes are expected to stabilize DNA in cold environments.

Among 25 *P. steinenii*-specific gene families, 14 gene families showed no similarity against protein databases and were considered lineage-specific, and the remaining 11 gene families categorized as *P. steinenii*-specific using Orthovenn2 (Supplementary Table 2) were distantly related to orthologous genes. These gene families could be grouped with other gene families sharing similar functions. These results indicated “phylogenetic instability,” defined as incongruence between the gene family phylogeny and the species tree. Phylogenetic instability can be caused by environmental changes during a speciation event and has been observed in genes related to responses to the environment, the immune response, and detoxification^29^. We further investigated the expanded acyl-CoA desaturase and Hsc70 gene families which have well-known functions. First, we used JBrowse^30^ to evaluate gene structures to verify the following: (1) whether each gene was located at independent positions in the assembled genome and (2) whether there is corresponding RNA evidence for the gene. Since all gene families in the acyl-CoA delta desaturase and Hsc70 families met these conditions, we conclude that they were naturally occurring genes in the *P. steinenii* genome.

Using dN/dS analysis, we inferred that positive selection by gene duplication occurred at the divergence branch between *P*. *steinenii*-specific and orthologous gene families in acyl-CoA delta desaturase and Hsc70. Tandem and dispersed duplications were identified as the major duplication types for these gene families, respectively. Gene duplication plays an important role in the emergence of new traits and physiological adaptations to extreme environmental conditions^31^. Positive selection events may promote favorable mutations in the context of rapid environmental changes^32^. In tandem duplication, a DNA segment is duplicated and inserted adjacent to the original segment, resulting in a structural rearrangement^33^. Dispersed duplication generates unpredictable and random patterns by DNA or RNA-based mechanisms, with two gene copies that are neither adjacent to each other in the genome nor within homologous chromosomal segments^34^. The species-specific 70 kilo Dalton heat-shock protein (Hsp70) family has been found in the genome of *Halicephalobus mephisto,* which lives in a warm fluid-filled aquifer of a South African gold mine^35^. Positive selection has occurred in several branches of Hsp70 to enable survival under thermal stress. Similar to *H. mephisto,* positive selection after duplication events in the *P. steinenii* genome may be a survival strategy to overcome severe low temperatures.

Acyl-CoA delta desaturase, also known as Stearoyl-CoA desaturase, is a fatty acid-modifying enzyme that catalyzes the insertion of a carbon–carbon double bond in saturated fatty acyl-CoA substrates, resulting in the desaturation of fatty acids. An increase in the proportion of unsaturated fatty acids reduces the phospholipid and fatty acid packing density, lowering the liquid to gel phase transition temperature and maintaining the function of the fluid bilayers even at low temperatures^31^. In addition, the expression of acyl-CoA delta desaturase affects the fatty acid composition of cholesterol esters^36^ and the observed gene family expansion may be related to cholesterol and lipoprotein homeostasis against extreme cold. Furthermore, the expanded acyl-CoA delta desaturase gene family is related to “defense response to Gram-positive bacterium”. We confirmed that nutrient-related GO terms (e.g., cellular response to nutrient levels, response to nutrient, response to fatty acid, and cellular response to nutrient) are associated with the acyl-CoA delta desaturase gene family, suggesting that this gene family plays an important role in the growth of *P. steinenii* as well as adaptation to cold environments. Indeed, previous studies have shown that essential polyunsaturated fatty acid (PUFA) synthesized by acyl-CoA delta desaturase, is positively correlated with the growth of marine shrimp^37^ and that PUFAs in insects (e.g., eicosapentaenoic acid and docosahexaenoic acid) influence larval growth and development^38^. Furthermore, we previously reported the presence of novel stearoyl-CoA desaturases in Antarctic marine copepod, suggesting that they serve as a physiological adaptation to maintain cellular membrane fluidity by increasing monounsaturated fatty acids^39^.

The two amino acid sequences of the expanded acyl-CoA delta desaturases showed positive selection at a BEB posterior probability threshold of 99% in the site-model. The positively selected amino acid residues of the expanded acyl-CoA delta desaturase were associated with the CoA binding site, providing a basis for further studies of adaptation to cold environment in Antarctica (Supplementary Fig. 3).

Rat SCD1, which performs as same role as acyl-CoA delta desaturase, has four transmembrane helices (TM1, TM2, TM3, and TM4), and residues 104 and 108 on TM2 are critical factors for the binding acyl chain length. Tyr104 is highly conserved in SCD1 of most animals, and rat SCD1 activity is highest for 17–19 acyl chains. However, if Threonine (as observed in ChDes1 from *Calanus hyperboreus*, a copepod in the northern Atlantic) replaces Tyrosine at the corresponding position, fatty acyl-CoA with 22–26 acyl chains is obtained, instead of fatty acyl-CoA with 18 acyl^13,40^. In the case of acyl-CoA delta desaturase orthologues, Tyr104 is present in rat SCD1 but various amino acids, such as Glycine, Alanine, Isoleucine, and Methionine, exist at the corresponding position in the expanded acyl-CoA delta desaturase gene family. Two genes (PS_09233 and PS_09234) among expanded acyl-CoA delta desaturaese genes had Isoleucine and Valine at the position corresponding to Ala108, identical to mutant rat SCD3 interacting with a relatively short fatty acyl-CoA with 14 acyl chains^13^, suggesting that they react with fatty acyl-CoA with 16 acyl chains (Supplementary Fig. 1). Thus, some of the expanded acyl-CoA delta desaturase genes had amino acids differences at the Tyr104 and Ala108 positions, suggesting that the chain length of fatty acyl-CoA binding to these genes may differ. The fatty acyl chain length control membrane fluidity^41^. Though somewhat speculative, these genes, which react with different acyl chain lengths of fatty acyl-CoA, are predicted to play a role regulating membrane fluidity to withstand cold environments in Antarctica.

The *P. steinenii*-specific Hsc70 family lacks the G/P-rich amino acid sequence Glu-Glu-Val-Asp (EEVD motif) in the C-terminal region of the substrate-binding domain. These regions are involved in the binding of co-chaperones and other heat shock proteins (Hsp) and the EEVD motif affects the ATPase activity of Hsc70. The EEVD motif is highly conserved in all eukaryotic Hsc70 and Hsp70 family members^14^, and loss of the EEVD motif weakens the chaperon function of Hsp70^42^. The amino acid sequence similarity of the substrate-binding domain was lower than that of the NBD (Supplementary Fig. 2). The chaperone function of Hsc70 in *P. steinenii* without the EEVD motif and G/P-rich region, and the low similarity in the SBD should be evaluated in further studies. Hsc70 is a constitutively expressed chaperone protein in most organisms, with important roles in physiological processes, such as protein folding and degradation, endocytosis and exocytosis, and autophagy^14^. It may also function as a thermal sensor; the unfolded protein binding ability of Hsc70 is temperature-dependent and is reduced under 30 °C^43^. In a study of familial cold autoinflammatory syndrome, the reduced binding ability of Hsc70 at low temperatures causes the hyperactivation of caspase-1 in NLRC4-H443P mutants^44^. For *P. steinenii* in Antarctica, at temperatures below 20 °C, functioning chaperone proteins as well as thermal sensors sensitive to different temperature ranges may be essential for survival. The expanded *P. steinenii*specific Hsc70 gene family showing phylogenetic instability could confer these functions.

We also attempted to identify common mechanisms underlying cold tolerance in *P. steinenii* and *B. antarctica*. GO enrichment analysis was performed for *P. steinenii* genes, which were included in gene families containing only *P. steinenii* and *B. antaractica* genes, against total *P. steinenii* genes. GO terms such as “O-glycan processing”, “trehalose transport”, “regulation of Toll signaling pathway”, and “tissue development” were enriched (Supplementary Fig. 4). The GO term “regulation of Toll signaling pathway” was derived from one gene family among the 68 rapidly evolving gene families, and similarities in proteins used for trehalose transport between *B. antarctica* and *P. steinenii* were very low (below 45%). These results were insufficient to support the existence of a shared mechanism for cold tolerance between these two species, and further analyses are required.

The winged midge *P. steinenii* survives in the harsh environments of Antarctica. To identify mechanisms contributing to cold adaptation, we obtained a high-quality assembled genome. Compared with the genomes of closely related dipteran species, 68 significantly rapidly evolving gene families including 25 *P. steinenii*-specific gene families were identified. These gene families are involved in the innate immune system, UPR, protein stability, unsaturated fatty acid metabolism, and DNA packaging. The extended acyl-CoA delta desaturase and Hsc70 gene families were found to contain the signatures of phylogenetic instability and positive selection following multiple gene duplication events. The expanded acyl-CoA delta desaturase gene family might be involved in membrane fluidity maintenance and nutrient-related responses in cold environments. Several positively selected amino acids were detected and differences in the amino acid sequences were predicted to determine fatty acyl chain lengths based on comparisons with orthologous gene families in mammals. The expanded Hsc70 gene family in *P. steinenii* lacks the C-terminal region and showed low sequence similarity in the SBD but had a conserved nucleotide-binding domain. Though we could not identify whether the functions of the *P. steinenii*-specific Hsc70 gene family were retained, the expansion of gene family might improve protein folding or thermal sensing in the cold environment. Our results suggest that *P. steinenii*, native to Antarctica, underwent gene family expansion via multiple gene duplications for adaptation to the cold environments.

## Methods

### Sample collection and sequencing

To obtain a high-quality *P. steinenii* genome, Oxford Nanopore long reads generated from a previous study were used^10^. *P. steinenii* samples were collected from a freshwater lake on King George Island, South Shetland Islands, Antarctica (62°14′ S, 58°47′ W) in January 2018 (Supplementary Fig. 5a,b). Total DNA was extracted from 50 adult midges using a DNeasy Tissue Kit (Qiagen, Valencia, CA, USA). All library construction and sequencing procedures using Oxford Nanopore technology were performed by Phyzen Co. Ltd. (Seongnam, Korea).

### Genome assembly

The Oxford Nanopore long reads and Illumina short reads obtained from a previous study of *P. steinenii*^9,10^ were assembled using NextDenovo (v.2.4.0) (https://github.com/Nextomics/NextDenovo) and polished using NextPolish (v.1.3.1)^16^ (Supplementary Table 4). The completeness of the assembled genome was evaluated using Benchmarking Universal Single-Copy Orthologs (v.5.1.3) (BUSCO)^45^.

### Repetitive sequence annotation

Repetitive sequences in the assembled genome were annotated using RepeatMasker (v.4.0.7)^46^. A de novo repeat library for the identification of repeat elements was constructed using RepeatModeler (v.1.0.11)^47^. The tRNA genes were identified using tRNAscan-SE (v. 2.0)^48^ with default parameter settings.

### RNA sequencing and transcriptome assembly

For RNA extraction, *P. steinenii* larvae collected at the same location as adults were transferred into conical tubes and acclimated at 4 °C at least 1 h prior to experiments. Thereafter, all *P. steinenii* larvae were divided into three temperature groups (4 °C, − 20 °C, and 20 °C) (Supplementary Fig. 5c). The larval samples in each group were exposed to the corresponding temperature for 30 min and then stored in RNAlater (Ambion, Inc., Austin, TX, USA) solution. The samples were immediately crushed in RNAlater using tissue grinder pestle. There were two (4 °C, and 20 °C) or three biological replicates (− 20 °C) for each treatment. Total RNA was extracted from 20 larvae in each group using the RNeasy Mini Kit (Qiagen) according to the manufacturer’s instructions. Total RNA was used for library construction and sequencing was performed on the Illumina HiSeq 2000 platform (Supplementary Table 5). The paired-end reads (151 bp × 2) obtained for total RNAs of *P. steinenii* samples were trimmed using FASTX-Toolkit (v.0.0.11) with the parameters “-t 30,” “-l 80,” and “-Q 33” and then assembled with reads (SRX1976254–SRX1976255) obtained from adults in a previous study^10^ using Trinity (v.2.11.0)^49^.

### Gene annotation

Protein-coding genes in the assembled genome were predicted using the MAKER annotation pipeline (v.2.31.10)^50^. AUGUSTUS (v.3.3.1)^51^ was used to predict gene loci in the *P. steinenii* genome. To increase the accuracy of gene prediction, protein homology evidence was obtained from five assembled genomes of dipteran species closely related to *P. steinenii*: *Aedes aegypti* (GCF_002204515, NCBI), *Anopheles gambiae* (GCF_000005575, NCBI), *B. antarctica* (ASM77530v1, Ensemble), *Clunio marinus* (GCA_90005825, NCBI), and *Drosophila melanogaster* (GCF_000001215, NCBI). Transcriptome assembly data were also used in the gene prediction process as expressed sequence tag (EST) evidence. The final gene model for *P. steinenii* was established by merging all gene models predicted by the above approaches. The annotation quality for the final gene model was assessed by verifying the gene completeness using BUSCO^45^. *P. steinenii* genes were annotated using Diamond BLASTp (v.2.0.8)^52^ against the EggNOG^53^ and KAAS^54^ databases.

### Gene ontology (GO) enrichment analysis

A Gene Ontology (GO) enrichment analysis of specific gene families was performed using the BiNGO (v.3.0.3) package^55^ in Cytoscape (v.3.7.2)^56^. Significantly enriched GO terms were validated using Fisher’s exact test and the *p* values were adjusted by the Benjamini & Hochberg procedure. Finally, significantly enriched GO terms for each gene were plotted using the stand-alone version of REVIGO (v.2015-02-17)^57^.

### Gene family analysis

OrthoVenn2 (https://orthovenn2.bioinfotoolkits.net)^12^ was used to identify orthologous genes across the *P. steinenii* assembled genome and five dipteran genomes (*A. aegypti*, *A. gambiae*, *B. antarctica*, *C. marinus*, and *D. melanogaster*), which were used in analyses of protein homology for gene prediction. Protein sequences from the six dipteran genomes were clustered with an E-value of 1e-5 and inflation value of 1.5. CAFE (v.4.2.1)^58^ was used with default parameters to identify rapidly evolving gene families among the orthologous gene families, which were confirmed using OrthoVenn2. The sequences of genes in orthologous gene families were aligned using MAFFT (v7.475)^59^ and the aligned sequences were used to construct a phylogenetic tree using FastTree (v.2.1.10)^60^ for CAFE input data. The divergence time between species was inferred using TimeTree^61^.

### Construction of phylogenetic trees based on acyl-CoA delta desaturase and Hsc70

Phylogenetic analyses of the *P. steinenii*-specific acyl-CoA delta desaturase and Hsc70 gene families were performed with orthologous gene families from six dipteran species (*A. aegypti*, *A. gambiae*, *B. antarctica*, *C. marinus*, *D. melanogaster,* and *P. steinenii*). All protein-coding gene sequences were aligned using PRANK (v.170427)^62^ with the “-DNA -codon” option and ambiguously aligned regions were eliminated using Gblocks (v.0.91b)^63^ with the “-t = c -e = -gb1 -b4 = 5 -d = y” option. The phylogenetic trees for the rapidly evolving gene families were also built using FastTree (v.2.1.10)^60^, and the orthologous gene family in *C. elegans* was designated as the outgroup.

### Analysis of sequence divergence in the acyl-CoA delta desaturase and Hsc70 gene families

To determine the level of divergence between *P. steinenii*-specific acyl-CoA delta desaturase and Hsc70 gene and orthologous gene families in other taxa due to gene duplication, *d*_N_/*d*_S_ (ω) values for these gene families were estimated using Codeml (PAML v.4.4). In the *d*_N_/*d*_S_ analysis (Supplementary Fig. 3), the null hypothesis (*H*_0_) was that *ω* is identical in *P. steinenii*-specific and orthologous gene families (ω_A0_ = ω_A1_ = ω_P1_ = ω_P0_; using model = 0 in Codeml). The alternative hypothesis (*H*_1_) was that *ω* differs between *P. steinenii*-specific and orthologous gene families (ω_A0_ = ω_A1_ ≠ ω_P1_ = ω_P0_; using model = 2 in Codeml). In addition, changes at sites under positive selection on the branch between *P. steinenii*-specific and orthologous gene families were estimated using different options in Codeml (Null model A: model = 2, NSsites = 2, fix_omega = 1; Model A: model = 2, NSsites = 2, fix_omega = 0, respectively). The *p *values were calculated using a likelihood ratio test (LRT) and adjusted using the false discovery rate (FDR).

### Structural analysis of the acyl-CoA delta desaturase gene family

For a detailed structural analysis of the expanded acyl-CoA delta desaturase family, multiple protein sequence alignments and gene structure predictions were conducted, using structurally well-known rat stearoyl-CoA desaturase protein sequences (SCD1) and acyl-CoA delta desaturase orthologues in the six dipteran genomes. The alignment and structure information for acyl-CoA delta desaturase gene families were obtained from Bai et al.^13^. Multiple sequence alignments were generated using ClustalW in BioEdit (v.7.2.5)^64^.

### Identification of gene duplications

The output of the all-versus-all Blast for the six dipteran genomes and the gff file were used as inputs for MCScanX (version Nov. 11, 2013)^65^. Then, ‘duplicate_gene_classifier’ was used to detect duplication types (e.g., singleton, dispersed, proximal, tandem, and whole genome/segmental duplication) with default settings.

## Supplementary Information

Supplementary Information 1.

Supplementary Information 2.

Supplementary Information 3.

## Data Availability

The raw sequencing data were deposited in NCBI Sequence Read Archive (SRA) with accessions SRX8008992, SRX8008993, SRX8008994, SRX8008995, SRX8008996, SRX8008997, SRX8008998, SRX8008999, and SRX8009000. The assembled genome sequence, protein sequence, transcript sequence, annotation information, Cuffdiff results, and Gene Ontology enrichment results for differentially expressed genes were deposited in Figshare^66^.

## References

[1] Kozeretska I, Serga S, Kovalenko P, Gorobchyshyn V, Convey P (2022). Belgica antarctica (Diptera: Chironomidae): A natural model organism for extreme environments. Insect Sci..

[2] Convey P (2010). Terrestrial biodiversity in Antarctica—Recent advances and future challenges. Polar Sci..

[3] Stork NE (2018). How many species of insects and other terrestrial arthropods are there on Earth?. Annu. Rev. Entomol..

[4] Teets NM (2019). Changes in energy reserves and gene expression elicited by freezing and supercooling in the Antarctic midge, *Belgica antarctica*. Insects.

[5] Shimada K, Ohyama Y, Pan C (1991). Cold-hardiness of the Antarctic winged midge *Parochlus steinenii* during the active season at King George Island. Polar Biol..

[6] Hahn S, Reinhardt K (2006). Habitat preference and reproductive traits in the Antarctic midge *Parochlus steinenii* (Diptera: Chironomidae). Antarct. Sci..

[7] Kelley JL (2014). Compact genome of the Antarctic midge is likely an adaptation to an extreme environment. Nat. Commun..

[8] Finch G (2020). Multi-level analysis of reproduction in an Antarctic midge identifies female and male accessory gland products that are altered by larval stress and impact progeny viability. Sci. Rep..

[9] Kim S (2017). Genome sequencing of the winged midge, *Parochlus steinenii*, from the Antarctic Peninsula. GigaScience.

[10] Shin SC (2019). Nanopore sequencing reads improve assembly and gene annotation of the *Parochlus steinenii* genome. Sci. Rep..

[11] Liu, H., Wu, S., Li, A. & Ruan, J. SMARTdenovo: A de novo assembler using long noisy reads. (2020). 10.46471/gigabyte.15 PMC9632051 36824332

[12] Xu L (2019). OrthoVenn2: A web server for whole-genome comparison and annotation of orthologous clusters across multiple species. Nucleic Acids Res..

[13] Bai Y (2015). X-ray structure of a mammalian stearoyl-CoA desaturase. Nature.

[14] Liu T, Daniels CK, Cao S (2012). Comprehensive review on the HSC70 functions, interactions with related molecules and involvement in clinical diseases and therapeutic potential. Pharmacol. Ther..

[15] Zhang J, Xie P, Lascoux M, Meagher TR, Liu J (2013). Rapidly evolving genes and stress adaptation of two desert poplars, *Populus euphratica* and *P. pruinosa*. PLoS ONE.

[16] Hu J, Fan J, Sun Z, Liu S (2020). NextPolish: a fast and efficient genome polishing tool for long-read assembly. Bioinformatics.

[17] Stokes BA, Yadav S, Shokal U, Smith L, Eleftherianos I (2015). Bacterial and fungal pattern recognition receptors in homologous innate signaling pathways of insects and mammals. Front. Microbiol..

[18] Lemaitre B, Hoffmann J (2007). The host defense of Drosophila melanogaster. Annu. Rev. Immunol..

[19] Tsakas S, Marmaras V (2010). Insect immunity and its signalling: an overview. Invertebr. Surviv. J..

[20] Weber AN (2003). Binding of the Drosophila cytokine Spätzle to Toll is direct and establishes signaling. Nat. Immunol..

[21] Valanne S, Wang J-H, Rämet M (2011). The Drosophila toll signaling pathway. J. Immunol..

[22] Lindsay SA, Wasserman SA (2014). Conventional and non-conventional Drosophila Toll signaling. Dev. Comp. Immunol..

[23] Sinclair BJ, Ferguson LV, Salehipour-Shirazi G, MacMillan HA (2013). Cross-tolerance and cross-talk in the cold: Relating low temperatures to desiccation and immune stress in insects. Integr. Comp. Biol..

[24] Salehipour-Shirazi G, Ferguson LV, Sinclair BJ (2017). Does cold activate the Drosophila melanogaster immune system?. J. Insect Physiol..

[25] Catalán TP, Wozniak A, Niemeyer HM, Kalergis AM, Bozinovic F (2012). Interplay between thermal and immune ecology: Effect of environmental temperature on insect immune response and energetic costs after an immune challenge. J. Insect Physiol..

[26] Hetz C, Papa FR (2018). The unfolded protein response and cell fate control. Mol. Cell.

[27] Chen Y, Brandizzi F (2013). IRE1: ER stress sensor and cell fate executor. Trends Cell Biol..

[28] Steiger D (2018). The serine/threonine-protein kinase/endoribonuclease IRE1α protects the heart against pressure overload-induced heart failure. J. Biol. Chem..

[29] Curran DM, Gilleard JS, Wasmuth JD (2018). MIPhy: Identify and quantify rapidly evolving members of large gene families. PeerJ.

[30] Buels R (2016). JBrowse: A dynamic web platform for genome visualization and analysis. Genome Biol..

[31] Zhang Z (2020). Adaptation to extreme Antarctic environments revealed by the genome of a sea ice green alga. Curr. Biol..

[32] Corona E, Dudley JT, Butte AJ (2010). Extreme evolutionary disparities seen in positive selection across seven complex diseases. PLoS ONE.

[33] Dong J, Qi M, Wang S, Yuan X (2020). Dintd: Detection and inference of tandem duplications from short sequencing reads. Front. Genet..

[34] Qiao X (2019). Gene duplication and evolution in recurring polyploidization–diploidization cycles in plants. Genome Biol..

[35] Weinstein DJ (2019). The genome of a subterrestrial nematode reveals adaptations to heat. Nat. Commun..

[36] Miyazaki M, Kim Y-C, Gray-Keller MP, Attie AD, Ntambi JM (2000). The biosynthesis of hepatic cholesterol esters and triglycerides is impaired in mice with a disruption of the gene for stearoyl-CoA desaturase 1. J. Biol. Chem..

[37] Uengwetwanit T (2021). A chromosome-level assembly of the black tiger shrimp (*Penaeus monodon*) genome facilitates the identification of growth-associated genes. Mol. Ecol. Resour..

[38] Li X (2022). Growth and fatty acid composition of black soldier fly *Hermetia illucens* (Diptera: Stratiomyidae) larvae are influenced by dietary fat sources and levels. Animals.

[39] Jung W, Kim EJ, Han SJ, Choi H-G, Kim S (2016). Characterization of stearoyl-CoA desaturases from a psychrophilic Antarctic Copepod, *Tigriopus kingsejongensis*. Mar. Biotechnol..

[40] Meesapyodsuk D, Qiu X (2014). Structure determinants for the substrate specificity of acyl-CoA Δ9 desaturases from a marine copepod. ACS Chem. Biol..

[41] Petkevicius K (2019). The Role of Macrophage Intracellular Lipid Partitioning in Glucose and Lipid Homeostasis During Obesity.

[42] Michels AA, Kanon B, Bensaude O, Kampinga HH (1999). Heat shock protein (Hsp) 40 mutants inhibit Hsp70 in mammalian cells. J. Biol. Chem..

[43] Leung S-M (1996). Thermal activation of the bovine Hsc70 molecular chaperone at physiological temperatures: Physical evidence of a molecular thermometer. Cell Stress Chaperones.

[44] Raghawan, A. K., Radha, V. & Swarup, G. HSC70 as a sensor of low temperature: Role in cold‐triggered autoinflammatory disorders. *FEBS J.* (2021). 10.1111/febs.16203 34535969

[45] Simão FA, Waterhouse RM, Ioannidis P, Kriventseva EV, Zdobnov EM (2015). BUSCO: assessing genome assembly and annotation completeness with single-copy orthologs. Bioinformatics.

[46] Chen N (2004). Using Repeat Masker to identify repetitive elements in genomic sequences. Curr. Protoc. Bioinform..

[47] Smit, A. F. & Hubley, R. RepeatModeler Open-1.0 http://www.repeatmasker.org (2008).

[48] Lowe TM, Eddy SR (1997). tRNAscan-SE: A program for improved detection of transfer RNA genes in genomic sequence. Nucleic Acids Res..

[49] Grabherr MG (2011). Trinity: reconstructing a full-length transcriptome without a genome from RNA-Seq data. Nat. Biotechnol..

[50] Cantarel BL (2008). MAKER: An easy-to-use annotation pipeline designed for emerging model organism genomes. Genome Res..

[51] Stanke M (2006). AUGUSTUS: Ab initio prediction of alternative transcripts. Nucleic Acids Res..

[52] Buchfink B, Xie C, Huson DH (2015). Fast and sensitive protein alignment using DIAMOND. Nat. Methods.

[53] Huerta-Cepas J (2016). eggNOG 45: A hierarchical orthology framework with improved functional annotations for eukaryotic, prokaryotic and viral sequences. Nucleic Acids Res..

[54] Moriya Y, Itoh M, Okuda S, Yoshizawa AC, Kanehisa M (2007). KAAS: an automatic genome annotation and pathway reconstruction server. Nucleic Acids Res..

[55] Maere S, Heymans K, Kuiper M (2005). BiNGO: a Cytoscape plugin to assess overrepresentation of gene ontology categories in biological networks. Bioinformatics.

[56] Shannon P (2003). Cytoscape: A software environment for integrated models of biomolecular interaction networks. Genome Res..

[57] Supek F, Bošnjak M, Škunca N, Šmuc T (2011). REVIGO summarizes and visualizes long lists of gene ontology terms. PLoS ONE.

[58] De Bie T, Cristianini N, Demuth JP, Hahn MW (2006). CAFE: A computational tool for the study of gene family evolution. Bioinformatics.

[59] Katoh K, Misawa K, Kuma KI, Miyata T (2002). MAFFT: A novel method for rapid multiple sequence alignment based on fast Fourier transform. Nucleic Acids Res..

[60] Price MN, Dehal PS, Arkin AP (2010). FastTree 2—Approximately maximum-likelihood trees for large alignments. PLoS ONE.

[61] Hedges SB, Dudley J, Kumar S (2006). TimeTree: A public knowledge-base of divergence times among organisms. Bioinformatics.

[62] Löytynoja A, Goldman N (2005). An algorithm for progressive multiple alignment of sequences with insertions. Proc. Natl. Acad. Sci..

[63] Talavera G, Castresana J (2007). Improvement of phylogenies after removing divergent and ambiguously aligned blocks from protein sequence alignments. Syst. Biol..

[64] Hall, T. In *Nucleic Acids Symposium Series* 95–98.

[65] Wang Y (2012). MCScanX: A toolkit for detection and evolutionary analysis of gene synteny and collinearity. Nucleic Acids Res..

[66] Shin SC, Lee H, Kim S (2022). figshare.

