# Supplementary material for: Gene family expansions in Antarctic winged midge as a strategy for adaptation to cold environments
Source: Sci Rep. 2022 Oct 29;12:18263. doi: 10.1038/s41598-022-23268-9 (PMC9617917; doi:10.1038/s41598-022-23268-9)
Supplement: Supplementary file 1 — Supplementary Information 1. [file 41598_2022_23268_MOESM1_ESM.docx]

Supplementary Information

**Gene family expansions in Antarctic winged midge as a strategy for adaptation to cold environments**

Heesoo Kim^1,2^, Han-Woo Kim^3,4^, Jun Hyuck Lee^3,4^, Joonho Park^5^, Hyoungseok Lee^1,4^, Sanghee Kim^1^*, Seung Chul Shin^1^*

^1^Division of Life Sciences, Korea Polar Research Institute (KOPRI), Incheon 21990, Republic of Korea

^2^Animal & Plant Research Department, Nakdonggang National Institute of Biological Resources (NNIBR)

^3^Research Unit of Cryogenic Novel Material, Korea Polar Research Institute, Incheon 21990, Korea

^4^Department of Polar Sciences, University of Science and Technology, Incheon 21990, Korea

^5^Department of Fine Chemistry, Seoul National University of Science and Technology, Seoul, South Korea.

The following materials include:

Supplementary Tables 1 to 5

Supplementary Figure 1 to 5


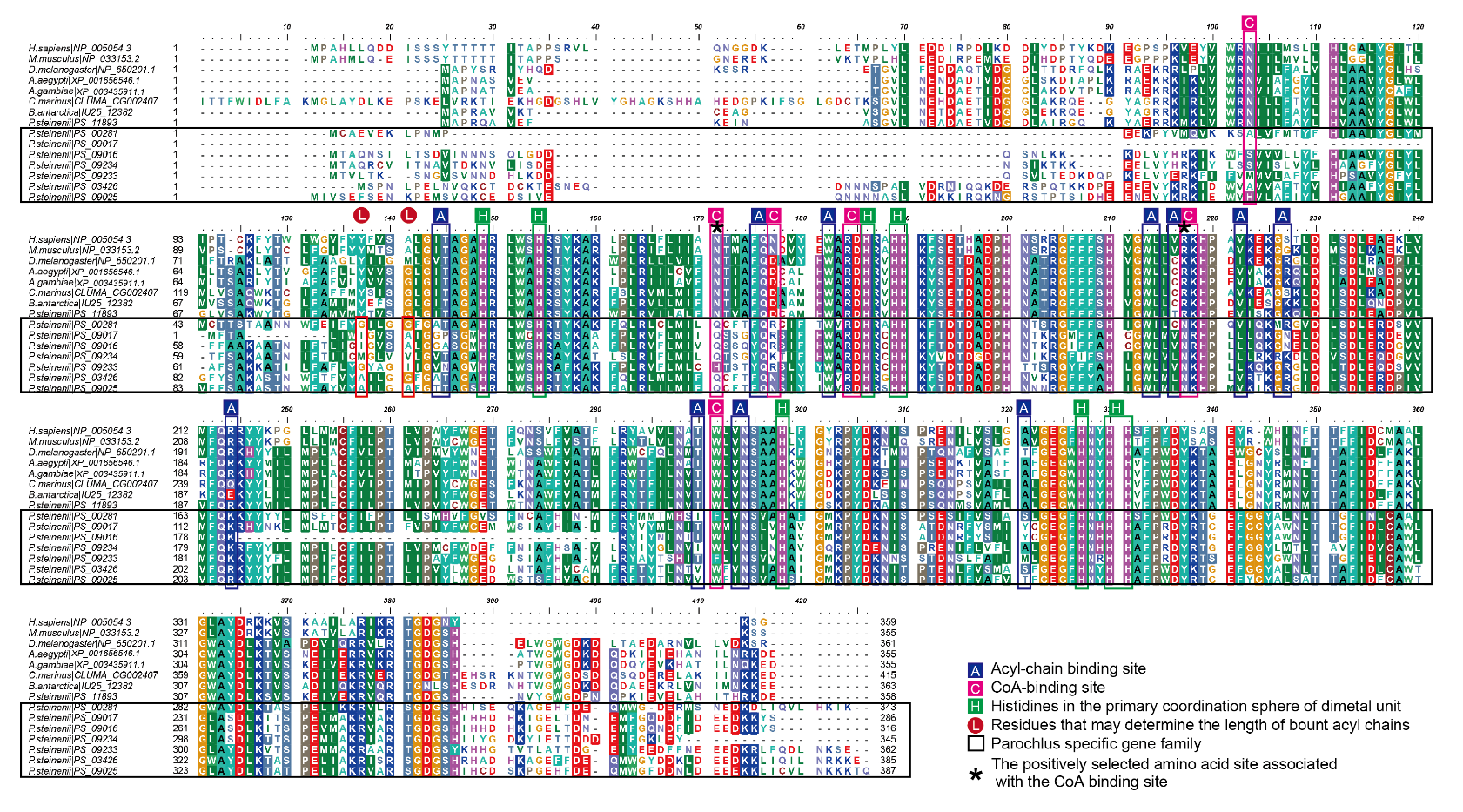


**Supplementary Figure 1.** Sequence alignment of the *P. steinenii*-specific acyl-CoA desaturase gene family with other integral membrane desaturases. The *P. steinenii*-specific gene family is outlined in black. Residues discussed in the text are highlighted in green (histidines in the primary coordination sphere of the dimetal unit), blue (acyl-chain binding site), purple (CoA binding site), and red (residues that may determine the length of bound acyl chains). The accession numbers for the sequences included in the alignment are as follows: mouse stearoyl-CoA desaturase (NP_033153.2), human stearoyl-CoA desaturase (NP_005054.3), *D. melanogaster* desaturase 2 (NP_650201.1), *A. aegypti* acyl-CoA delta desaturase isoform X2 (XP_001656546.1), *A. gambiae* acyl-CoA delta desaturase (XP_003435911.1), and *C. marinus* (CLUMA_CG002407). *B. antarctica* (IU25_12382) delta-9 desaturase, acyl-CoA desaturase sequences of PS_11893 in group2588 and PS_00281, PS_09017, PS_09016, PS_09234, PS_09233, PS_03426, and PS_09025 in group1599 from OrthoVenn2 results were included in this alignment. The positively selected amino acid sites associated with the CoA binding site are marked with asterisks.

*

*

*

*

*

*

*


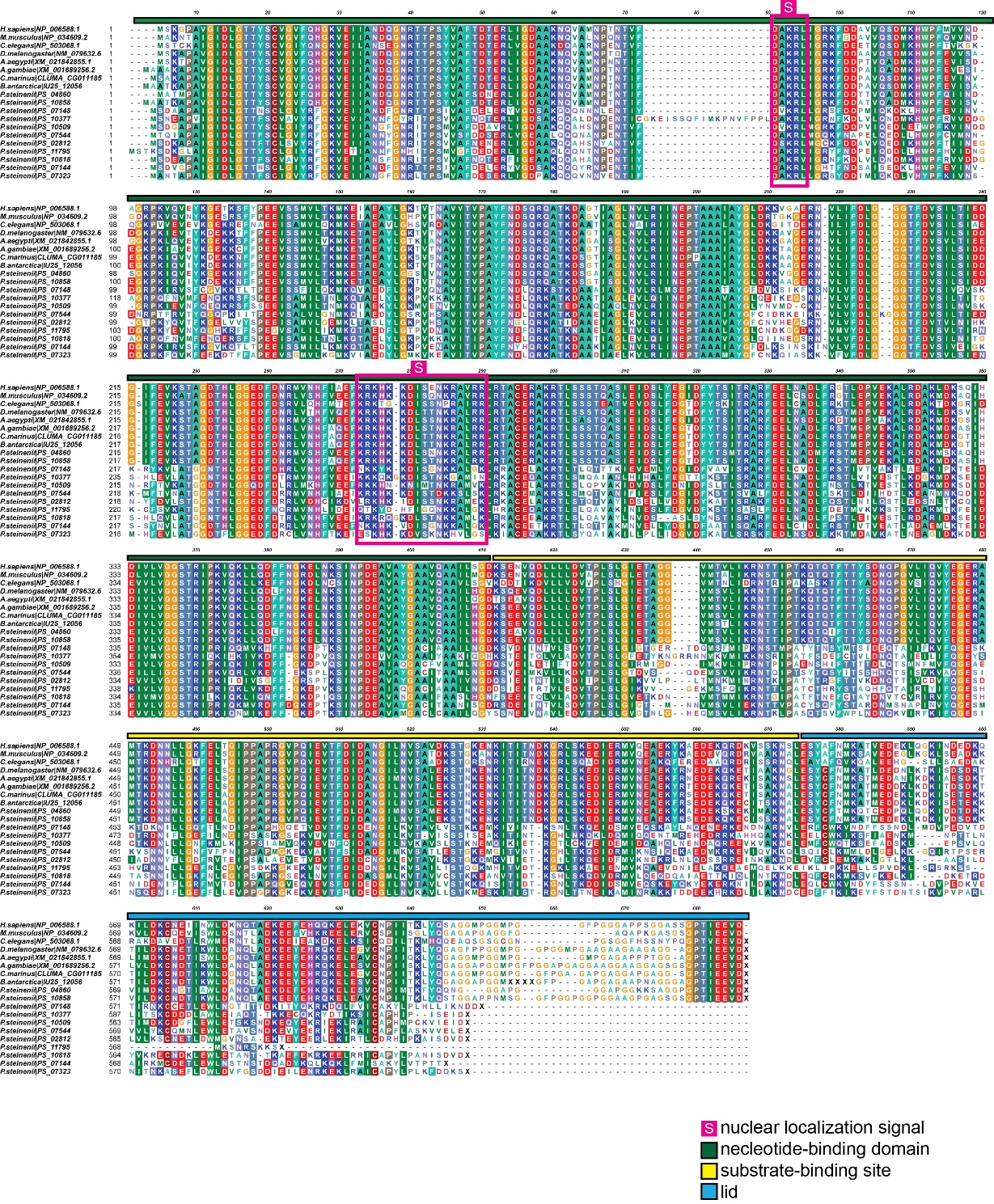


The positively selected amino acid site

*

*

*

*

*

*

*

*

*

*

*

*

*

*

*

*

*

*

**Supplementary Figure 2.** Sequence alignment of the *P. steinenii*-specific Hsc70 gene family with other Hsc70 proteins. The *P. steinenii*-specific proteins are outlined in black. The regions corresponding to the nucleotide-binding domain, substrate-binding site, and lid region are indicated by green, yellow, and blue bars, respectively. Nuclear localization signal sequences are highlighted in purple. The accession numbers for sequences included in this alignment are as follows: human heat shock cognate 71 kDa protein isoform 1 (NP_0065881.1), mouse heat shock 70 kDa protein 1A (NP_034609.2), *C. elegans* heat shock protein Hsp-1 (NP_503068.1). *D. melanogaster* heat shock protein cognate 4 (NP_001262586.1), *A. aegypti* heat shock protein cognate 4 (XP_021698547.1), *A. gambiae* acyl-CoA delta desaturase (XP_001689308.2), and *C. marinus* (CLUMA_CG011185). *B. antarctica* (IU25_12056) delta-9 desaturase. Protein sequences of PS_04860 and PS_10858 for group441 and protein sequences of PS_07148, PS_10377, PS_10509, PS_07544, PS_02812, PS_11795, PS_10818, PS_07144, and PS_07323 in group513 from OrthoVenn2 results were included in this alignment. The positively selected amino acid sites marked with asterisks.


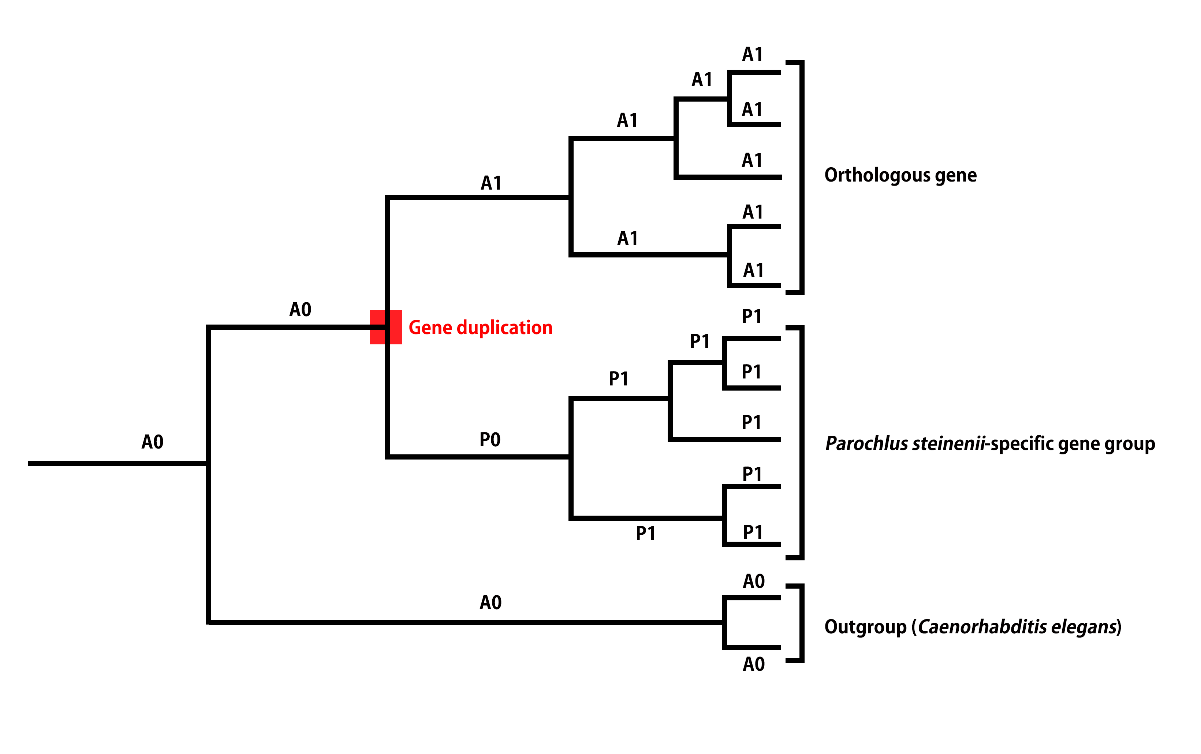


**Supplementary Figure 3.** Hypothesis for detecting positive selection. The null hypothesis (H0) is that ω is identical in *P. steinenii*-specific and orthologous gene families (ωA0 = ωA1 = ωP1 = ωP0; using model = 0 in Codeml). The alternative hypothesis (H1) is that ω differs between *P. steinenii*-specific and orthologous gene families (ωA0 = ωA1 ≠ ωP1 = ωP0; using model = 2 in Codeml).


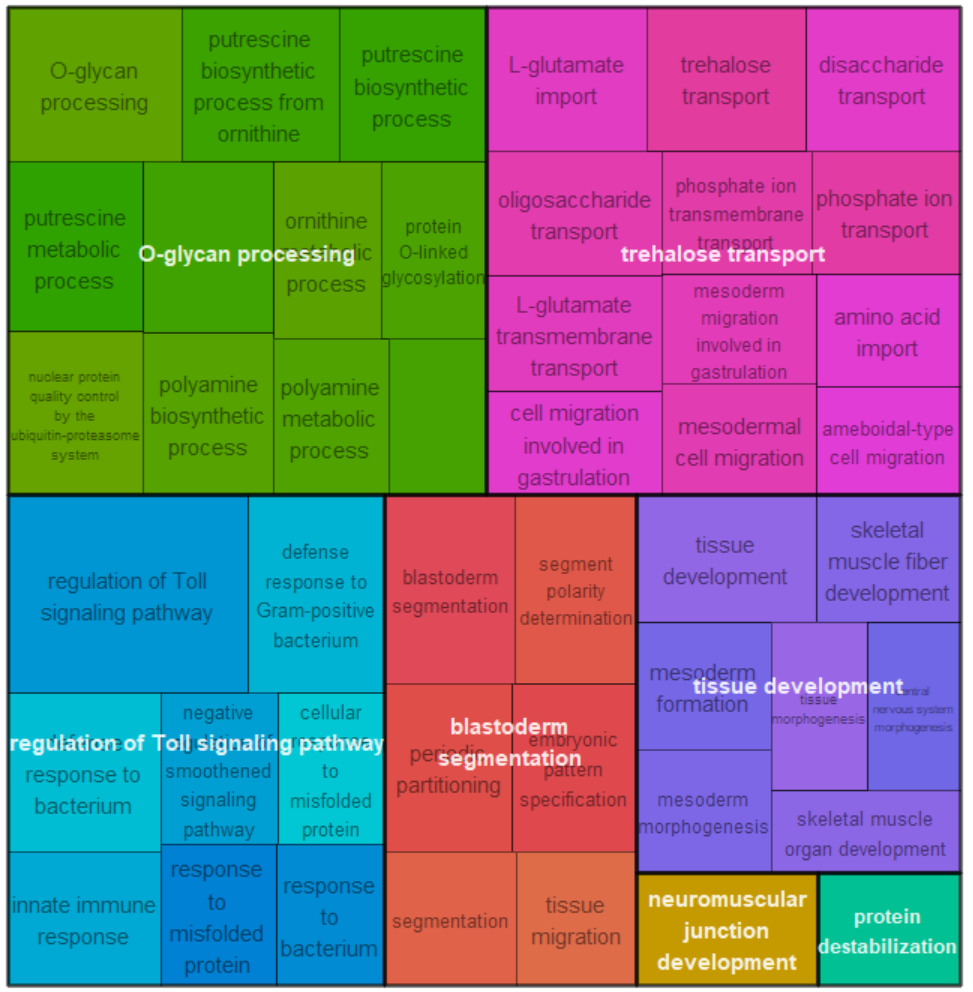


**Supplementary Figure 4.** Enriched GO terms among gene families including only *P. steinenii* and *B. antarctica* proteins. GO enrichment analysis was performed for *P. steinenii* genes, which were included in gene families containing only *P. steinenii* and *B. antaractica* genes, against total *P. steinenii* genes. Enriched biological process terms were visualized using REVIGO.


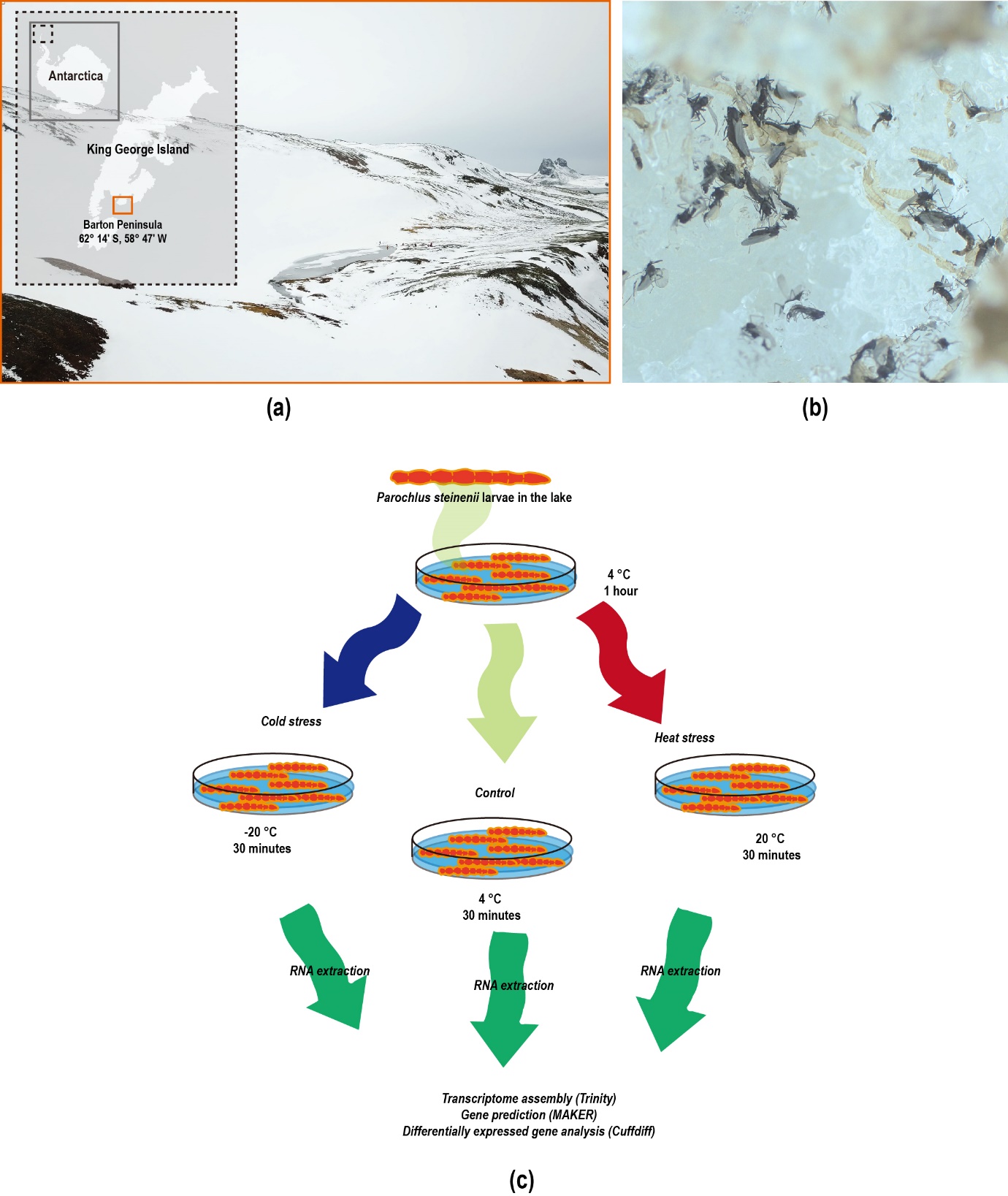


**Supplementary Figure 5.** Collection and analysis of *P. steinenii.* (a) Freshwater lake formed from melting ice located in King George Island. (b) *P. steinenii* adults residing in ice. (c) A schematic overview of the transcriptome analysis

**Supplementary Table 1.** Repeat prediction using the *de* *novo* RepeatModeler repeat library and the number of tRNAs

| **Repeat elements** | | **# of element** | **Length (%)** |
| --- | --- | --- | --- |
| Retrotransposon | SINE | 93 | 57,765 (0.04%) |
|  | LINE | 1,567 | 947,132 (0.66%) |
|  | LTR | 696 | 1,314,035 (0.91%) |
| DNA elements | | 2,345 | 678,668 (0.47%) |
| Unclassified elements | | 29,910 | 13,109,506 (9.09%) |
| Small RNA | | 247 | 308,697 (0.21%) |
| Tandem repeat | Satellites | 321 | 69,242 (0.05%) |
|  | Simple repeats | 27,315 | 1,200,741 (0.83%) |
| Low complexity | | 9,125 | 426,902 (0.30%) |
| tRNA | | 492 | - |

**Supplementary Table 2.** List of 68 rapidly evolving gene families in *P. steinenii* with *p* < 0.05 determined by CAFÉ*.* Gene number in each insect species and the duplicated gene origin of *P. steinenii* are indicated. “*P. steinenii-*specific gene family” denotes the gene family which includes only *P. steinenii* gene based on the OrthoVenn2 analysis, and “lineage-specific gene” denotes unique gene families in *P. steinenii*.

| Number | OrthoVenn2 | Predicted function | *Parochlus steinenii* | *Belgica antarctica* | *Aedes aegypti* | *Anopheles gambiae* | *Clunio marinus* | *Drosophila melanogaster* | Sum | *P. steinenii*-specific gene family | lineage-specific gene | dispersed duplication | Proximal duplication | Tandem duplication | WGD or segmental |
| --- | --- | --- | --- | --- | --- | --- | --- | --- | --- | --- | --- | --- | --- | --- | --- |
| 1 | group02 | leucine-rich domain | 57 | 0 | 0 | 0 | 0 | 0 | 57 |  |  | 21 | 16 | 20 | 0 |
| 2 | group03 | Serine protease | 37 | 1 | 2 | 1 | 2 | 3 | 37 |  |  | 23 | 6 | 8 | 0 |
| 3 | group05 | Trypsin-like serine protease, Brachcyurin | 14 | 4 | 7 | 13 | 3 | 0 | 14 |  |  | 5 | 2 | 7 | 0 |
| 4 | group08 | mobile element jockey reverse transcriptase | 22 | 5 | 1 | 1 | 4 | 1 | 22 |  |  | 22 | 0 | 0 | 0 |
| 5 | group18 | histone H4 | 11 | 1 | 0 | 8 | 8 | 0 | 22 |  |  | 2 | 9 | 0 | 0 |
| 6 | group20 | unknown function | 27 | 0 | 1 | 0 | 0 | 0 | 27 |  |  | 21 | 2 | 4 | 0 |
| 7 | group21 | serine protease venom protease | 20 | 1 | 1 | 1 | 1 | 3 | 20 |  |  | 6 | 3 | 11 | 0 |
| 8 | group35 | zinc finger protein | 23 | 0 | 0 | 0 | 0 | 0 | 23 |  |  | 3 | 5 | 13 | 2 |
| 9 | group36 | aminopeptidase N | 13 | 2 | 3 | 2 | 1 | 2 | 13 |  |  | 0 | 1 | 12 | 0 |
| 10 | group38 | unknown function | 23 | 0 | 0 | 0 | 0 | 0 | 23 | ● | ○ | 9 | 8 | 6 | 0 |
| 11 | group41 | helicase | 8 | 3 | 6 | 1 | 2 | 2 | 8 |  |  | 5 | 0 | 3 | 0 |
| 12 | group47 | c-type lectin | 19 | 1 | 0 | 0 | 1 | 0 | 19 |  |  | 3 | 0 | 16 | 0 |
| 13 | group49 | zinc finger protein | 18 | 2 | 0 | 1 | 0 | 0 | 18 |  |  | 4 | 2 | 12 | 0 |
| 14 | group50 | serine threonine protein kinase | 17 | 1 | 1 | 1 | 0 | 1 | 17 |  |  | 10 | 1 | 6 | 0 |
| 15 | group51 | pickpocket protein 28 | 21 | 0 | 0 | 0 | 0 | 0 | 21 | ● |  | 8 | 0 | 13 | 0 |
| 16 | group52 | unknown function | 21 | 0 | 0 | 0 | 0 | 0 | 21 | ● | ○ | 10 | 8 | 2 | 0 |
| 17 | group60 | histone H3 | 9 | 1 | 0 | 7 | 3 | 0 | 18 |  |  | 1 | 4 | 0 | 4 |
| 18 | group62 | cuticle protein | 12 | 3 | 1 | 0 | 3 | 0 | 12 |  |  | 2 | 2 | 8 | 0 |
| 19 | group70 | farnesol dehydrogenase | 9 | 1 | 0 | 1 | 6 | 1 | 9 |  |  | 6 | 2 | 0 | 1 |
| 20 | group72 | unknown function | 18 | 0 | 0 | 0 | 0 | 0 | 18 | ● | ○ | 9 | 6 | 3 | 0 |
| 21 | group78 | IRE-1 like protein | 17 | 0 | 0 | 0 | 0 | 0 | 17 |  |  | 6 | 3 | 8 | 0 |
| 22 | group80 | unknown function | 17 | 0 | 0 | 0 | 0 | 0 | 17 |  |  | 13 | 4 | 0 | 0 |
| 23 | group83 | unknown function | 14 | 2 | 0 | 0 | 0 | 1 | 14 |  |  | 13 | 1 | 0 | 0 |
| 24 | group87 | unknown function | 17 | 0 | 0 | 0 | 0 | 0 | 17 | ● | ○ | 4 | 3 | 8 | 2 |
| 25 | group93 | leucine rich domain | 16 | 0 | 0 | 0 | 0 | 0 | 16 |  |  | 5 | 0 | 11 | 0 |
| 26 | group100 | cuticle protein | 7 | 1 | 0 | 0 | 0 | 7 | 7 |  |  | 1 | 0 | 6 | 0 |
| 27 | group111 | unknown function | 15 | 0 | 0 | 0 | 0 | 0 | 15 | ● | ○ | 15 | 0 | 0 | 0 |
| 28 | group119 | unknown function | 12 | 1 | 0 | 0 | 1 | 0 | 12 |  |  | 3 | 1 | 8 | 0 |
| 29 | group160 | unknown function | 13 | 0 | 0 | 0 | 0 | 0 | 13 | ● | ○ | 3 | 1 | 8 | 0 |
| 30 | group175 | unknown function | 11 | 0 | 0 | 0 | 1 | 0 | 11 |  |  | 4 | 3 | 4 | 0 |
| 31 | group177 | terminal uridylyl transferases like protein | 10 | 1 | 0 | 0 | 1 | 0 | 11 |  |  | 2 | 6 | 2 | 0 |
| 32 | group252 | modular serine protease | 10 | 1 | 0 | 0 | 0 | 0 | 10 |  |  | 8 | 0 | 2 | 0 |
| 33 | group270 | unknown function | 11 | 0 | 0 | 0 | 0 | 0 | 11 |  |  | 3 | 0 | 7 | 0 |
| 34 | group331 | phenoloxidase-activating factor (trypsin) | 7 | 2 | 0 | 0 | 1 | 0 | 7 |  |  | 0 | 1 | 6 | 0 |
| 35 | group360 | Astacin | 8 | 0 | 0 | 0 | 2 | 0 | 8 |  |  | 3 | 2 | 3 | 0 |
| 36 | group366 | cathepsin L1-like protein | 10 | 0 | 0 | 0 | 0 | 0 | 10 | ● |  | 4 | 1 | 5 | 0 |
| 37 | group367 | serine protease trypsin | 10 | 0 | 0 | 0 | 0 | 0 | 10 | ● |  | 1 | 4 | 5 | 0 |
| 38 | group435 | unknown function | 7 | 0 | 0 | 1 | 0 | 1 | 7 |  |  | 5 | 0 | 2 | 0 |
| 39 | group471 | zinc finger protein | 8 | 1 | 0 | 0 | 0 | 0 | 8 |  |  | 2 | 4 | 2 | 0 |
| 40 | group474 | unknown function | 9 | 0 | 0 | 0 | 0 | 0 | 9 |  |  | 9 | 0 | 0 | 0 |
| 41 | group511 | unknown function | 7 | 0 | 0 | 0 | 2 | 0 | 7 |  |  | 4 | 3 | 0 | 0 |
| 42 | group513 | hsc70 | 9 | 0 | 0 | 0 | 0 | 0 | 9 | ● |  | 5 | 2 | 2 | 0 |
| 43 | group522 | leucine rich domain | 9 | 0 | 0 | 0 | 0 | 0 | 9 | ● | ○ | 1 | 0 | 8 | 0 |
| 44 | group523 | unknown function | 9 | 0 | 0 | 0 | 0 | 0 | 9 | ● | ○ | 3 | 0 | 6 | 0 |
| 45 | group524 | G-protein coupled receptor Mth2 | 9 | 0 | 0 | 0 | 0 | 0 | 9 | ● |  | 1 | 0 | 8 | 0 |
| 46 | group525 | unknown function | 9 | 0 | 0 | 0 | 0 | 0 | 9 | ● |  | 1 | 2 | 6 | 0 |
| 47 | group584 | clavesin | 8 | 0 | 0 | 0 | 0 | 0 | 8 |  |  | 1 | 0 | 7 | 0 |
| 48 | group719 | zinc finger protein | 8 | 0 | 0 | 0 | 0 | 0 | 8 |  |  | 0 | 2 | 6 | 0 |
| 49 | group778 | unknown function | 8 | 0 | 0 | 0 | 0 | 0 | 8 |  |  | 1 | 0 | 7 | 0 |
| 50 | group813 | unknown function | 4 | 0 | 4 | 0 | 0 | 0 | 4 |  |  | 4 | 0 | 0 | 0 |
| 51 | group814 | carbohydrate binding | 8 | 0 | 0 | 0 | 0 | 0 | 8 | ● |  | 6 | 0 | 2 | 0 |
| 52 | group1143 | zinc finger protein | 7 | 0 | 0 | 0 | 0 | 0 | 7 |  |  | 0 | 0 | 7 | 0 |
| 53 | group1405 | zinc finger protein | 6 | 1 | 0 | 0 | 0 | 0 | 6 |  |  | 2 | 1 | 3 | 0 |
| 54 | group1494 | unknown function | 7 | 0 | 0 | 0 | 0 | 0 | 7 |  |  | 3 | 1 | 2 | 1 |
| 55 | group1573 | unknown function | 5 | 0 | 0 | 0 | 2 | 0 | 5 |  |  | 5 | 0 | 0 | 0 |
| 56 | group1575 | serine protease persephone | 6 | 0 | 1 | 0 | 0 | 0 | 6 |  |  | 1 | 0 | 5 | 0 |
| 57 | group1579 | Penelope-like elements and similar proteins | 7 | 0 | 0 | 0 | 0 | 0 | 7 |  |  | 3 | 2 | 2 | 0 |
| 58 | group1598 | unknown function | 7 | 0 | 0 | 0 | 0 | 0 | 7 | ● | ○ | 7 | 0 | 0 | 0 |
| 59 | group1599 | acyl-CoA delta desaturase | 7 | 0 | 0 | 0 | 0 | 0 | 7 | ● |  | 2 | 0 | 5 | 0 |
| 60 | group6037 | c-type lection | 6 | 0 | 0 | 0 | 0 | 0 | 6 |  |  | 2 | 0 | 4 | 0 |
| 61 | group6072 | unknown function | 6 | 0 | 0 | 0 | 0 | 0 | 6 | ● | ○ | 0 | 2 | 4 | 0 |
| 62 | group6073 | unknown function (c-type lectin) | 6 | 0 | 0 | 0 | 0 | 0 | 6 | ● | ○ | 2 | 1 | 3 | 0 |
| 63 | group6074 | unknown function | 6 | 0 | 0 | 0 | 0 | 0 | 6 | ● | ○ | 6 | 0 | 0 | 0 |
| 64 | group6075 | UDP-glucuronosyltransferase | 6 | 0 | 0 | 0 | 0 | 0 | 6 | ● |  | 3 | 3 | 0 | 0 |
| 65 | group6076 | sodium-coupled monocarboxylate transporter | 6 | 0 | 0 | 0 | 0 | 0 | 6 | ● |  | 2 | 0 | 4 | 0 |
| 66 | group6077 | unknown function | 6 | 0 | 0 | 0 | 0 | 0 | 6 | ● | ○ | 1 | 0 | 5 | 0 |
| 67 | group6078 | fatty acid hydroxylase domain-containing protein 2 | 6 | 0 | 0 | 0 | 0 | 0 | 6 | ● |  | 0 | 0 | 6 | 0 |
| 68 | group6079 | unknown function | 6 | 0 | 0 | 0 | 0 | 0 | 6 | ● | ○ | 6 | 0 | 0 | 0 |

**Supplementary Table 3.** Comparison of duplicate gene types determined using the MCScanX tool for six dipteran insect genomes

|  | *Parochlus steinenii* | *Belgica antarctica* | *Drosophila melanogaster* | *Aedes aegypti* | *Anopheles gambiae* | *Clunio marinus* |
| --- | --- | --- | --- | --- | --- | --- |
| Singleton | 3,824 (30.6%) | 4,917 (36.4%) | 21,649 (70.5%) | 17,421 (61.5%) | 5,820 (41.3%) | 13,646 (59.9%) |
| Dispersed repeat | 5,599 (45.0%) | 7,079 (52.4%) | 6,151 (20.0%) | 7,310 (25.8%) | 5,489 (38.9%) | 6,564 (28.8%) |
| Proximal repeat | 842 (6.8%) | 4,21 (3.1%) | 1,364 (4.4%) | 1,166 (4.1%) | 619 (4.4%) | 1,153 (5.2%) |
| Tandem repeat | 2,092 (16.8%) | 1,093 (8.1%) | 1,539 (5.0%) | 2,345 (8.3%) | 2,174 (15.4%) | 1,404 (6.2%) |
| WGD or segmental repeat | 104 (0.8%) | 0 | 0 | 62 (0.2%) | 0 | 0 |
| sum | 12,461 | 13,510 | 30,703 | 28,304 | 14,102 | 22,767 |

*WGD denotes whole genome duplication

**Supplementary Table 4.** Summary of nanopore read statistics. kbp = kilobase pairs. Raw data was base-called using Guppy software (v3.1.5).

|  | Raw data |
| --- | --- |
| SRA experiment | SRX5001002 |
| Total read number | 1,999,088 |
| Total read bases (bp) | 10,970,289,711 |
| Mean read length (bp) | 5487.61 (10.4) |
| Max length (bp) | 96,705 |
| Read length N50 | 12,381 |
| Number above 5kbp/total length (bp)/percentage of the total reads (%) | 692,507/8,819,419,598/80 |
| Number above 5kbp/total length (bp)/percentage of the total reads (%) | 378,620/6,548,956,539/60 |
| Number above 5kbp/total length (bp)/percentage of the total reads (%) | 101,037/2,638,003,734/24 |

**Supplementary Table 5.** Summary of the RNA-seq read statistics obtained in this study.

| SRA experiment | Temperature (°C) | Time | Number of larvae (each) | Total length sum (bases) | Total read number | Read length (bases) |
| --- | --- | --- | --- | --- | --- | --- |
| SRX8008992 | 4 | 30min | 20 | 2680260570 | 17750070 | 151 |
| SRX8008993 | 4 | 30min | 20 | 2557358650 | 16936150 | 151 |
| SRX8008996 | 20 | 30min | 20 | 3006644352 | 19911552 | 151 |
| SRX8008997 | 20 | 30min | 20 | 2393026860 | 15847860 | 151 |
| SRX8008998 | -20 | 30min | 20 | 2254102632 | 14927832 | 151 |
| SRX8008999 | -20 | 30min | 20 | 2718174254 | 18001154 | 151 |
| SRX8009000 | -20 | 30min | 20 | 2586165222 | 17126922 | 151 |
|  |  |  |  |  |  |  |
